# Comparative adjuvant therapy to reduce vancomycin-associated nephrotoxicity in an animal model

**DOI:** 10.1128/aac.00887-26

**Published:** 2026-09-10

**Authors:** Yongqi Xiao, Nicholas S. Teran, Cole S. Hudson, Masayuki Nigo, Vincent H. Tam

**Affiliations:** 1 Department of Pharmacy Practice and Translational Research, University of Houston College of Pharmacy 15507, Houston, Texas, USA; 2 Division of Infectious Diseases, Department of Medicine, Houston Methodist Hospital, Texas Medical Center 23534, Houston, Texas, USA; University of California San Francisco, San Francisco, California, USA

**Keywords:** glycopeptide, nephroprotectant, acute kidney injury

## Abstract

Vancomycin is a mainstay antibiotic for serious gram-positive infections, but nephrotoxicity remains a major barrier to its optimal use. In this study, we compared the effectiveness of montelukast and zileuton as adjuvant strategies to reduce vancomycin-associated nephrotoxicity. Montelukast only had a marginal effect in vancomycin-treated animals, whereas zileuton significantly delayed nephrotoxicity onset. These findings support further formulation and dose-optimization studies to repurpose zileuton as a renal-protective adjuvant.

## INTRODUCTION

Vancomycin is one of the most widely utilized intravenous antibiotics for the treatment of infections due to gram-positive bacteria, particularly in the management of serious infections due to methicillin-resistant *Staphylococcus aureus* (1). Vancomycin-associated nephrotoxicity poses a significant challenge limiting optimal dosing (2). An expert consensus guideline recommended the transition from vancomycin trough-based to area-under-the-curve (AUC_24 hours_ 400–600 mg·h/L)-based dosing to optimize the pharmacokinetic predictor for efficacy while minimizing toxicities (1). Despite the revised dosing strategies and avoidance of concomitant nephrotoxins as the mainstay approach for reducing vancomycin-associated nephrotoxicity, this adverse event remains significant and is associated with morbidity, mortality, and increased healthcare costs (3). As a result, identifying strategies to ameliorate the nephrotoxicity associated with vancomycin remains a paramount priority.

Previously, we demonstrated that concomitant montelukast was associated with reduced prevalence of vancomycin-associated acute kidney injury (AKI) in a retrospective study (4). Patients receiving montelukast were noted to have a fourfold reduction in AKI prevalence after adjusting for independent risk factors, including weight and intensive care unit admission. Another independent investigator group has also reported a significantly lower risk of AKI and hemodialysis in a large cohort study of propensity-matched patients (*n* = 283,550), with the use of montelukast in patients receiving vancomycin (5). However, limitations in the applicability of adjuvant montelukast have been described, including its utility as an acute rescue agent, as our patient cohort all received montelukast prior to vancomycin exposure and neuropsychiatric concerns leading to a boxed warning by the Food and Drug Administration (6, 7). At the same time, our group investigated the protective effects of an alternative agent, zileuton, in the attenuation of renal toxicity observed with aminoglycosides and polymyxins (8). We speculated that the benefits could be extended to vancomycin. To facilitate the development of a first-in-class therapy for renal protection, the objective of this study was to compare the effectiveness of montelukast to zileuton in reducing vancomycin-associated nephrotoxicity in an animal model.

Montelukast powder (USP) was obtained from MilliporeSigma (St. Louis, MO). Zileuton powder (USP) was obtained from Supelco (Bellefonte, PA). Vancomycin hydrochloride (USP) was obtained from Slate Run Pharmaceuticals (Columbus, OH). Piccolo comprehensive metabolic panels were purchased from Abaxis (Union City, CA). Sprague Dawley male (325–350 g) and female (225–250 g) rats were purchased from Envigo (Indianapolis, IN).

Experimental details of the animal model of nephrotoxicity associated with vancomycin were previously described (8). The animals received food and water *ad libitum* and were kept on a 12-hour light/dark cycle. All animals (both sexes) were given intraperitoneal vancomycin (200 mg/kg once daily) over 10 days and randomly divided into three groups. Fifteen minutes prior to vancomycin administration, the control group was given saline (*n* = 20); the second group was given subcutaneous montelukast (30 mg/kg, *n* = 10); and the last group was given intraperitoneal zileuton (4 mg/kg, *n* = 10). Blood samples (approximately 200 µL) were collected from the tail tip at baseline and daily; serum samples were obtained by centrifugation and assayed for creatinine concentrations. Nephrotoxicity endpoint was defined as doubling of serum creatinine concentration from baseline. The onset of nephrotoxicity in various treatment cohorts was compared using a time-to-event (e.g., Kaplan–Meier) analysis and log-rank test. Statistical analyses were performed using GraphPad Prism 11 (GraphPad Software, Boston, MA). *P* values ≤0.05 were considered significant.

**Fig 1:**
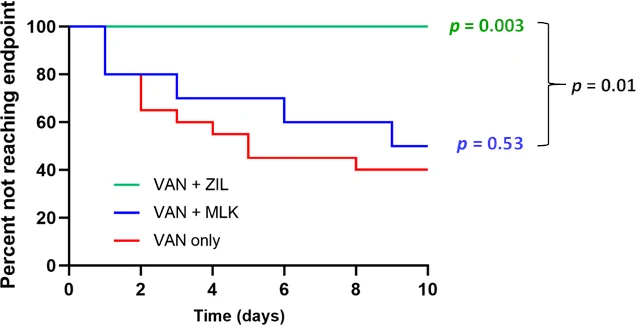
Onset of vancomycin-associated nephrotoxicity in rats. MLK, montelukast; VAN, vancomycin; ZIL, zileuton. *n* = 20 for VAN alone and *n* = 10 for all other groups. Kaplan–Meier survival curves were compared using the log-rank test. Compared with VAN alone, *P* = 0.003 for VAN + ZIL and *P* = 0.53 for VAN + MLK. Direct comparison of VAN + ZIL versus VAN + MLK yielded *P* = 0.01.

In the control group (i.e., vancomycin was given alone without any adjuvant therapy), 60% of animals developed nephrotoxicity by day 10; the median time to nephrotoxicity was 5 days. Compared with the control group, montelukast only marginally delayed the onset of nephrotoxicity (median time to nephrotoxicity was 9.5 days, *P* = 0.53), whereas zileuton significantly delayed nephrotoxicity (*P* = 0.003). When two treatment groups were compared directly, zileuton also significantly delayed the onset of nephrotoxicity relative to montelukast (*P* = 0.01) (Fig. 1). No apparent sex-related differences in nephrotoxicity or treatment response were observed in this study. The renal protective effect was subsequently confirmed by histopathological studies, in which zileuton markedly attenuated the extent of vancomycin-induced tubulointerstitial injury, with only mild tubular dilatation observed (9).

In this study, we directly compared the effectiveness of montelukast to zileuton in alleviating vancomycin-associated nephrotoxicity in an established animal model. Since the need for vancomycin for treating acute infections is often unpredictable, pre-treatment with a renal-protective agent is not always feasible. Therefore, an adjuvant strategy that can be initiated concomitantly with vancomycin would be more practical clinically. Without prior exposure to either adjuvant, zileuton provided greater protection than montelukast when administered together with vancomycin.

Montelukast and zileuton are both indicated for asthma treatment by modulating leukotriene-mediated pathways. Leukotrienes are inflammatory mediators; they promote bronchoconstriction and airway inflammation in the lungs. Mechanistically, montelukast blocks cysteinyl leukotriene receptors, whereas zileuton acts upstream as a 5-lipoxygenase inhibitor to reduce leukotriene formation (10, 11). Together with our findings, this suggests that broader inhibition of leukotriene production may provide stronger protection against vancomycin-associated renal injury. In contrast, the protective association observed with montelukast in retrospective clinical studies may reflect chronic exposure, which allows leukotriene receptor blockade to be more fully established before renal injury occurs (4, 5). Of note, exposure to both agents has been associated with the Nrf2/Keap1 antioxidant pathway, resulting in increased expression of heme-oxygenase-1 in cell culture models (4, 9). Moreover, zileuton has been shown to attenuate kidney injury caused by other insults, including chemotherapy and ischemia, further supporting its broader potential as a renal-protective adjuvant (12, 13).

Initial dose selection was empiric and primarily based on animal tolerance (and drug solubility). Using allometric scaling, we expected systemic exposure of montelukast to be higher than that observed after a typical 10 mg oral dose in humans (14). In contrast, systemic zileuton exposure was considered comparable to typical oral dosing in humans (i.e., 600–1,200 mg), according to subsequent pharmacokinetic studies (15). Therefore, zileuton may offer a wider safety margin for dose escalation, if needed.

Several limitations should be considered. This proof-of-concept study evaluated only a single dose of each adjuvant, precluding assessment of dose-dependent effects. Pharmacokinetic/pharmacodynamic analyses were not performed to guide optimal dosing. In addition, nephrotoxicity was defined using serum creatinine only, which may not fully capture early kidney injury.

In conclusion, our data support the greater potential of zileuton as an adjuvant to attenuate vancomycin-associated nephrotoxicity. This would allow the optimal use of vancomycin for serious infections. Formulation and dose optimization studies are ongoing to repurpose zileuton as a renal-protective agent.
